# Dating the Age of the SIV Lineages That Gave Rise to HIV-1 and HIV-2

**DOI:** 10.1371/journal.pcbi.1000377

**Published:** 2009-05-01

**Authors:** Joel O. Wertheim, Michael Worobey

**Affiliations:** Department of Ecology and Evolutionary Biology, University of Arizona, Tucson, Arizona, United States of America; University of Auckland, New Zealand

## Abstract

Great strides have been made in understanding the evolutionary history of simian immunodeficiency virus (SIV) and the zoonoses that gave rise to HIV-1 and HIV-2. What remains unknown is how long these SIVs had been circulating in non-human primates before the transmissions to humans. Here, we use relaxed molecular clock dating techniques to estimate the time of most recent common ancestor for the SIVs infecting chimpanzees and sooty mangabeys, the reservoirs of HIV-1 and HIV-2, respectively. The date of the most recent common ancestor of SIV in chimpanzees is estimated to be 1492 (1266–1685), and the date in sooty mangabeys is estimated to be 1809 (1729–1875). Notably, we demonstrate that SIV sequences sampled from sooty mangabeys possess sufficient clock-like signal to calibrate a molecular clock; despite the differences in host biology and viral dynamics, the rate of evolution of SIV in sooty mangabeys is indistinguishable from that of its human counterpart, HIV-2. We also estimate the ages of the HIV-2 human-to-human transmissible lineages and provide the first age estimate for HIV-1 group N at 1963 (1948–1977). Comparisons between the SIV most recent common ancestor dates and those of the HIV lineages suggest a difference on the order of only hundreds of years. Our results suggest either that SIV is a surprisingly young lentiviral lineage or that SIV and, perhaps, HIV dating estimates are seriously compromised by unaccounted-for biases.

## Author Summary

HIV/AIDS continues to be a major health problem worldwide. An understanding of the evolution of HIV in humans may be greatly improved by detailed knowledge of its predecessor, simian immunodeficiency virus (SIV), in non-human primates. While HIV causes AIDS in humans, SIV generally produces a benign infection in its natural hosts. This avirulence is often attributed to coevolution between the virus and its host, possibly due to codivergence over millions of years. Here, we provide a temporal reference for evolution of SIV in its natural primate hosts. Using state-of-the-art molecular clock dating techniques, we estimate the time of most recent common ancestor for SIV in sooty mangabeys and chimpanzees at 1809 (1729–1875) and 1492 (1266–1685), respectively. These ages indicate that SIV may have infected these natural hosts for only hundreds of years before giving rise to HIV. This short duration suggests that viral–host coevolution over millions of years is not a likely explanation for the widespread avirulence of SIV. Finally, despite differences between SIV and HIV in host biology and viral pathogenicity, we have found clear and direct evidence that SIV evolves at a rapid rate in its natural hosts, an evolutionary rate that is indistinguishable from that of HIV in humans.

## Introduction

HIV/AIDS is the result of at least eleven cross-species transmission events of simian immunodeficiency virus (SIV) from non-human African primates to humans. Three transmissions of SIVcpz from the central African chimpanzee subspecies (*Pan troglodytes troglodytes*) gave rise to HIV-1 groups M, N and O [1], and the other eight SIVsm transmissions from sooty mangabeys (*Cercocebus torquatus atys*) gave rise to HIV-2 groups A through H [2],[3]. All three HIV-1 groups, plus HIV-2 groups A and B, have established human-to-human transmission chains, with HIV-1 group M causing pandemic HIV/AIDS. The six other HIV-2 lineages do not appear to be transmissible among humans [2].

Determining when the virus jumped into humans has been a priority for HIV researchers. By analyzing viral sequences obtained over several decades and calibrating a molecular clock based on observed nucleotide changes, a reliable rate of sequence evolution can be inferred. Korber *et al.* used this method to estimate the time of most recent common ancestor (tMRCA) for HIV-1 group M at 1931 (1915–1941) [4]; this estimate has recently been pushed back slightly to 1908 (1884–1924) [5]. The tMRCA of HIV-1 group O was estimated to be 1920 (1890–1940) [6]. Both HIV-1 group M and O dates were inferred using a relaxed molecular clock, which allows the rate of evolution to vary along different branches of the tree. HIV-2 group A and B tMRCAs were estimated to be 1940 (1924–1956) and 1945 (1931–1959), respectively [7]. These dates were estimated using a strict molecular clock, (i.e., a single, constant evolutionary rate along all branches). No estimate currently exists for the tMRCA of HIV-1 group N.

There has also been success in locating the populations of chimpanzees and sooty mangabeys whose SIVs are the direct ancestors of the transmissible HIV lineages (i.e., the SIVs that lie basal to HIV-1 and HIV-2 on the SIV/HIV phylogeny). Extensive non-invasive fecal sampling of wild chimpanzees pointed to the origin of HIV-1 group M in southeastern Cameroon and HIV-1 group N in south central Cameroon [8]. Although a reciprocally monophyletic clade of SIVcpz has been found in the eastern chimpanzee subspecies (*Pan troglodytes schweinfurthii*), virus from this group does not appear to have jumped successfully into humans [9]. Surprisingly, the SIV lineage that falls immediately basal to HIV-1 group O was found in gorillas, suggesting that they might have been an intermediate host between chimpanzees and humans [10]. Similar fecal analysis in sooty mangabeys indicated that HIV-2 groups A and B were likely transmitted to humans in Côte d'Ivoire [11].

Despite these findings, an important question about the origins of SIV/HIV remains unanswered: How long have these primate hosts been infected with SIV? Answering this question would help determine the length of time SIV was in sooty mangabeys and chimpanzees before giving rise to the transmissible HIV lineages. It might also shed light on the tMRCA of the dozens of other SIV lineages.

Determining the age of SIV would provide perspective on the spread of the virus among African primate species and the subsequent zoonoses. Knowing the age may also have implications for the evolution of pathogenicity and virulence in HIV. AIDS-like symptoms have rarely been observed in non-human African primates infected with SIV [12],[13]. Historically, this lack of disease was attributed to the codivergence and coevolution of SIV and their primate hosts over millions of years [14] (we use the term codivergence instead of cospeciation, because codivergence considers phylogenetic congruence irrespective of species classification, whereas cospeciation implies that SIVs infecting different primates can be classified as species complexes). Although there is significant correspondence between the SIV and host phylogenies, detailed analysis of this relationship suggested that a preferential host switching model, in which cross-species transmissions of SIV are more likely to occur between closely related primates, could account for this correspondence [15]. Furthermore, subsequent analysis of SIV infecting various African green monkey species, thought to be exemplary of codivergence, demonstrated a lack of evidence for host-virus codivergence [16]. In addition, the codivergence hypothesis does not account for the observation that SIV is geographically confined and naturally infects only African primates. Finally, even with biologically unrealistic assumptions about a molecular clock, Sharp *et al.* were unable to push the tMRCA of all SIV beyond 2500 years [17]. If it were demonstrated that SIV has evolved in a clock-like manner, then we might be able to accurately determine the age of SIV.

Here, we use relaxed molecular clock phylogenetic inference to determine the tMRCA of SIVsm/HIV-2 and SIVcpz/HIV-1. We also provide, to our knowledge, the first estimate of the age of HIV-1 group N. Taken together, these dates suggest that SIV may indeed be a relatively young viral clade and that its transmission into humans is a natural process.

## Results

### Dating SIVsm/HIV-2 tMRCAs

**Figure 1:**
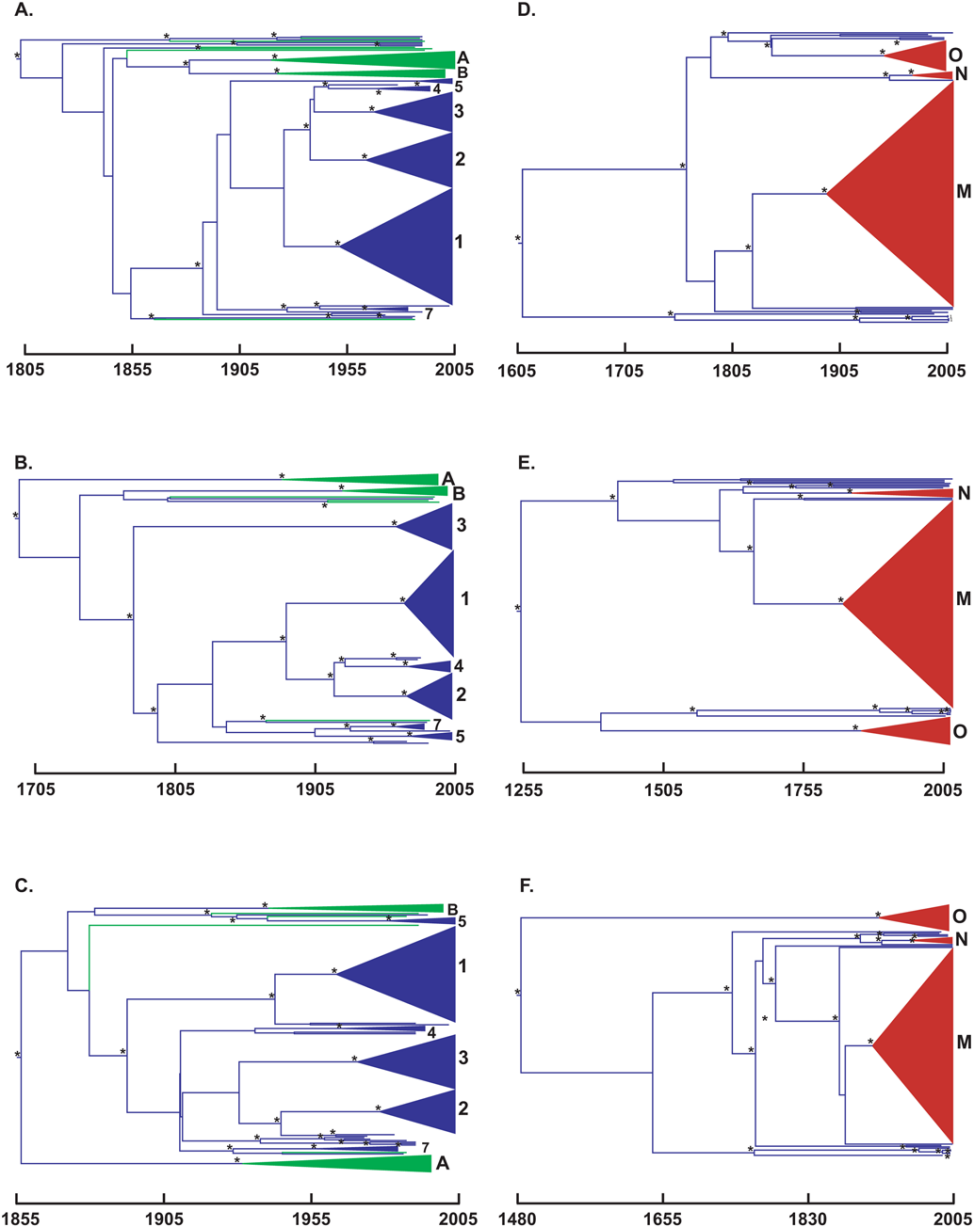
Maximum clade credibility trees. SIVsm/HIV-2 loci (A) *gag*, (B) *pol*, and (C) *env* and SIVcpz/HIV-1 loci (D) *gag*, (E) *pol*, and (F) *env* are depicted. SIV lineages are blue, HIV-2 lineages are green, and HIV-1 lineages are red. Major SIVsm clades are designated by number and the HIV clades are designated by group. Nodes with posterior probability >0.9 are indicated with an asterisk.

We inferred phylogenies for SIVsm/HIV-2 *gag*, *pol*, and *env* loci under a relaxed molecular clock in a Bayesian Markov chain Monte Carlo (BMCMC) framework (Figure 1A–C). In each tree there was very high posterior support for monophyly in HIV-2 group A, HIV-2 group B, and the major SIVsm clades identified by Apetrei *et al.* [18]. The position of the root, determined by the BMCMC analysis, was also highly supported in each of the three trees. Phylogenetic inference using the three loci produces different topologies, which was expected given the observation of recombination by Apetrei *et al.* in their initial analysis of these loci in SIVsm [18].

The tMRCA estimates for the root of the SIVsm/HIV-2 trees differed as well (Table 1). The *pol* locus had the oldest root, putting the tMRCA of SIVsm/HIV-2 at 1686 (1525–1811). Estimates from *gag* and *env* were significantly younger, placing the SIVsm/HIV-2 tMRCA at 1809 (1798–1875) and 1861 (1788–1915), respectively. Although *gag* was older than *env*, this difference was not significant. The *pol* results also indicated older dates than *gag* and *env* for the tMRCA of HIV-2 groups A and B, although these differences were not significant. With the exception of the *env* tMRCA estimate for HIV-2 group A, all three genes suggested a slightly older origin of both HIV-2 groups A and B than previously reported by Lemey *et al.* [7]. There were no discernable differences in the tMRCA estimates of these three genes for the major SIVsm clades (Table 2), although there were significant differences in the age of deeper SIVsm coalescent events among SIVsm groups 1, 2, 3, 4, and 7 [P(*gag*<*pol*) = 0.003; P(*env*<*pol*)<0.001)].

**Table 1:** tMRCA dates of SIVsm/HIV-2 and SIVcpz/HIV-1 clades and comparisons between loci.

Clade	*gag* tMRCA (95% HPD)	*pol* tMRCA (95% HPD)	*env* tMRCA (95% HPD)	P(*gag*<pol)	P(gag<*env*)	P(*env*<*pol*)
SIVsm	1809 (1729–1875)	1686 (1525–1811)	1861 (1788–1915)	0.050	0.870	0.012
HIV-2 A	1932 (1906–1955)	1905 (1857–1949)	1942 (1921–1959)	0.150	0.720	0.060
HIV-2 B	1935 (1907–1961)	1914 (1868–1955)	1937 (1914–1958)	0.210	0.522	0.171
SIVcpz	1618 (1471–1746)	1265 (658–1679)	1492 (1266–1685)	0.051	0.159	0.212
HIV-1 M	1912 (1887–1935)	1795 (1627–1900)	1894 (1857–1927)	0.005	0.218	0.033
HIV-1 N	1966 (1953–1977)	1932 (1876–1968)	1963 (1948–1977)	0.032	0.416	0.053
HIV-1 O	1942 (1922–1958)	1827 (1680–1917)	1905 (1866–1938)	<0.001	0.024	0.062

**Table 2:** tMRCA dates and comparisons between SIVsm/HIV-2 and SIVsm-only analyses.

Locus	Clade	SIVsm/HIV-2 tMRCA (95% HPD)	SIVsm-only tMRCA (95% HPD)	P (SIVsm/HIV-2>SIVsm-only)
*gag*	SIVsm	1814 (1731–1878)	1745 (1586–1879)	0.777
	SIVsm-1	1959 (1935–1977)	1973 (1964–1981)	0.112
	SIVsm-2	1966 (1951–1978)	1969 (1955–1980)	0.350
	SIVsm-3	1962 (1944–1977)	1958 (1929–1978)	0.590
	SIVsm-4	1980 (1974–1985)	1982 (1977–1986)	0.316
	SIVsm-5	1981 (1968–1993)	1982 (1972–1991)	0.485
	SIVsm-7	1967 (1958–1975)	1966 (1954–1976)	0.526
*pol*	SIVsm	1697 (1543–1815)	1461 (971–1835)	0.825
	SIVsm-1	1971 (1960–1980)	1973 (1962–1983)	0.362
	SIVsm-2	1975 (1966–1984)	1976 (1967–1985)	0.431
	SIVsm-3	1973 (1956–1986)	1974 (1956–1985)	0.464
	SIVsm-4	1975 (1968–1982)	1976 (1969–1983)	0.418
	SIVsm-5	1972 (1947–1989)	1973 (1950–1990)	0.446
	SIVsm-7	1971 (1964–1976)	1971 (1963–1976)	0.497
*env*	SIVsm	1880 (1844–1916)	1894 (1852–1927)	0.293
	SIVsm-1	1968 (1958–1977)	1965 (1951–1977)	0.645
	SIVsm-2	1982 (1974–1988)	1979 (1971–1986)	0.694
	SIVsm-3	1972 (1960–1983)	1969 (1955–1981)	0.599
	SIVsm-4	1967 (1958–1975)	1966 (1955–1975)	0.553
	SIVsm-5	1982 (1969–1994)	1977 (1963–1989)	0.714
	SIVsm-7	1968 (1960–1974)	1967 (1957–1974)	0.558

### Dating SIVcpz/HIV-1 tMRCAs

We inferred phylogenies for SIVcpz/HIV-1 *gag*, *pol*, and *env* loci under a relaxed molecular clock in a BMCMC framework (Figure 1D–F). There was very high posterior support for monophyly in each of the three HIV-1 lineages as well as for the position of the root. The three loci produced different topologies, which is not surprising given the recombinant history of HIV-1 group N [1],[19].

Like in the previous SIVsm/HIV-2 analyses, the three loci produced variable tMRCA estimates for the root and the major HIV-1 lineages (Table 1). Again, *pol* had the oldest dates, with the SIVcpz/HIV-1 tMRCA at 1265 (658–1679). In contrast, *gag* had the youngest date at 1618 (1471–1746), and *env* produced an intermediate date at 1492 (1266–1685). tMRCA estimates from *gag* and *env* for both HIV-1 group M and HIV-1 group O agreed with previous estimates from Worobey *et al.* [5] and Lemey *et al.* [6], although the *pol* tMRCA dates were nearly twice as old. There was good agreement between *gag* and *env* when dating HIV-1 group N, placing the tMRCAs at 1966 (1953–1977) and 1963 (1948–1977), respectively. We also performed additional phylogenetic inference to ensure that we captured the deepest available HIV-1 group N lineages in our analyses (Figure S1).

### Resolving the discrepancy among tMRCA estimates

There were significant discrepancies among the tMRCA estimates from *gag*, *pol*, and *env* in both the SIVsm/HIV-2 and SIVcpz/HIV-1 analyses. We initially thought that this discordance was due to recombination among the loci. If recombination were responsible, the different tMRCA estimates would actually represent different times of coalescence. When examining the phylogenies, however, we found very little evidence for this scenario. There were highly similar patterns of diversity in the SIVsm clades and in the HIV-1 group M sub-types among the three loci. An explanation of recombination would necessitate selective sweeps in *gag* and *env*, which would then go on to recreate the ancestral diversity seen in the *pol* phylogeny. For example, HIV-1 group M would have a mean tMRCA around 1795, and, approximately 100 years later, part of the genome would have experienced a selective sweep that gave rise to the same pattern of sub-type diversity (Table 1).

We then explored the possibility that some of these analyses were biased. That this discrepancy among tMRCA estimates was most pronounced in the older nodes indicated a loss of signal due to this bias deeper in the phylogeny. We examined the demographic parameters (e.g. population size or growth rate) from the three loci in the SIVsm/HIV-2 and SIVcpz/HIV-1 analyses. There were significant differences in these parameter estimates from *gag* to *pol* and from *env* to *pol* (P<0.05). Even though these genes evolved along different topologies, their demographic history, and therefore the demographic parameters inferred from them, should be the same. We hypothesized that some genes lack sufficient demographic signal to draw accurate inference about tMRCAs and that allowing the three loci to combine their demographic signal might homogenize their tMRCA estimates.

To test this hypothesis, we compared a partition analysis where the concatenated genes shared a single demographic scenario to analyses where that scenario was inferred for each gene independently. This analysis was performed separately for SIVsm/HIV-2 and SIVcpz/HIV-1 under two different coalescent scenarios: constant population size and exponential growth. In all cases, allowing the three loci to share demographic information homogenized the tMRCA estimates such that there were no longer significant differences in the age of the root among the phylogenies. Among the SIVsm/HIV-2 loci, *gag* tMRCA estimates change the least between the partition analysis and the analyses where demographic parameters were inferred for each gene independently (Table 3). Since tMRCA estimates from g*ag* are the most robust to combining demographic parameters, these dates should be taken as the best estimates. Among the SIVcpz/HIV-1 loci, *env* produced the most stable tMRCA estimates, changing as little as 0.01% under the exponential growth model (Table 3). This finding suggests that *env* provided the best tMRCA estimates for SIVcpz/HIV-1.

**Table 3:** Percent change of root tMRCA estimates from single-gene to partition analysis.

Taxa	Coalescent Model	Change from Single-Gene to Partition Analysis		
		*gag*	*pol*	*Env*
SIVsm/HIV-2	Constant	−6.8%	11.0%	−28.5%
	Exponential	−14.5%	42.4%	−38.7%
SIVcpz/HIV-1	Constant	−12.2%	5.1%	0.01%
	Exponential	−13.2%	4.0%	2.5%

Given the different selective regimes that these loci experienced, it is unlikely that the differences in the tMRCA estimates among the three loci were due entirely to variable demographic signal. Nevertheless, accounting for this variation in demographic signal appears to have resolved the majority of the discrepancy among the tMRCA estimates. In addition, even if the differences among the tMRCA estimates were real, and due to recombination, all three loci suggest root ages that are of the same order of magnitude. Therefore, although we discuss these results with reference to what appear to be the most robust loci (*gag* for SIVsm/HIV-2 and *env* for SIVcpz/HIV-1), we would be able to draw the same general conclusions from any of the three loci. Finally, we emphasize that although the tMRCA estimates presented include the mean of the posterior distribution, this mean estimate is meaningful only in context of the 95% highest probability density (HPD).

### Clock-like signal in SIV

We next sought to determine if the dates we obtained were the result of clock-like signal within SIVsm or whether SIVsm had no clock-signal and we were inadvertently extrapolating HIV-2 rates across the entire tree. We compared the date estimates from *gag*, *pol*, and *env* to analyses where all non-SIVsm sequences were excluded. For all three genes, there were no significant differences in the tMRCAs between the full and SIVsm-only datasets in any of the clades measured, including all SIVsm (Table 2). Furthermore, there was no significant difference between the SIVsm *gag* substitution rate we estimated of 1.38×10^−3^ (1.03×10^−3^–1.73×10^−3^) substitutions/site/year and the HIV-2 group A substitution rate of 1.22×10^−3^ substitutions/site/year estimated by Lemey *et al.* [7]. This similarity indicates that SIVsm does indeed have sufficient clock-like signal to date tMRCAs, and it does not appear to evolve at a different rate than HIV-2 group A, despite differences in host biology and pathogenicity.

BMCMC analysis of an alignment containing only SIVcpz did not provide meaningful date estimates, as the tMRCA estimates from these runs were indistinguishable from the prior distribution of tMRCA estimates. Therefore, the tMRCA date we inferred for SIVcpz may have incorporated HIV-1 rates that could be biasing this estimate. However, our previous analysis of SIVsm/HIV-2 suggested that HIV-2 rates did not appreciably affect SIVsm tMRCA estimates.

### Coalescent scenarios in SIVsm

In a population of constant size, the most basal lineages are consistently lost due to normal coalescent processes; the age of the root is expected to be approximately two times the effective population size [20]. However, if the population is expanding exponentially, the basal lineages will be maintained until a carrying capacity is reached. The BMCMC method used here provides a convenient framework in which to test whether a constant size or exponential growth model better describes the dynamics of a population: If the 95% HPD of the exponential growth rate excludes zero, then a constant population size can be strongly rejected. To determine if exponential growth explains the SIVsm population dynamics better than a constant population size, we looked at the exponential growth rate in alignments containing only SIVsm sequences.

Exponential growth rate 95% HPDs from *gag* and *env* in the SIVsm analysis excluded zero; however, the growth rate 95% HPD from *pol* did not exclude zero. Nevertheless, the exponential growth rate 95% HPD estimated in partition analysis (combining demographic signal from all three loci) rejected a constant population size. Thus, it seems probable that *pol* failed to reject a constant population size because it simply lacked sufficient demographic signal. Therefore, it is likely that SIVsm has not been evolving at a constant population size for the past 200 years.

### Maximum age of HIV-1 groups M and N

As a result of the discovery of SIVcpz lineages that are very closely related to HIV-1 groups M and N [8], we were able to investigate when HIV-1 groups M and N shared a most recent common ancestor (MRCA) with an SIVcpz lineage. Prior to our study, there existed one estimate of this date for HIV-1 group M and SIVcpz at 1675 (1590–1761) [21]; however, this date was obtained using only two SIVcpz sequences, neither of which lies directly basal to HIV-1 group M. Our *env* analysis suggested that HIV-1 group M and the SIVcpz sequence that lies immediately basal to it shared an MRCA in 1853 (1799–1904), and HIV-1 group N and its sister SIVcpz shared a MRCA in 1921 (1885–1955). These dates represent the maximum age for the introduction of HIV-1 groups M and N into humans.

### Time before zoonoses

We determined the number of years between the SIVsm and SIVcpz tMRCAs and those of the five transmissible HIV lineages (Table 4). If the SIVsm and SIVcpz tMRCAs represent the time SIV has been infecting each host, then this estimate would tell us the number of years that SIV was present in sooty mangabeys and chimpanzees before jumping into humans and giving rise to the transmissible lineages of HIV. We note, however, that a tMRCA estimate will tend to post-date the actual introduction of viral lineages into a new host if genetic diversity has since been lost or is not fully sampled. We believe such comparisons still provide useful information as long as this caveat is recognized. The times between the root of the SIVsm/HIV-2 tree and the base of the HIV-2 group A clade and the group B clade were 122.8 (57.2–199.9) and 126.2 (59.2–203.7) years, respectively. The time between the SIVcpz root and the HIV-1 lineages was 402.8 (231.0–601.4) years for HIV-1 group M, 471.6 (291.6–693.2) years for HIV-1 group N, and 413.5 (247.1–621.3) years for HIV-1 group O. These estimates are from the *gag* locus for SIVsm/HIV-2 and from the *env* locus for SIVcpz/HIV-1; partition analyses indicated that these genes were the most reliable for each clade. Ninety-five percent HPD intervals are larger for these estimates than for other single clades because the age estimates for any two clades are not perfectly correlated.

**Table 4:** Years between SIVsm and SIVcpz roots and the tMRCA of HIV lineages.

Clade	*gag* years (95% HPD)	*pol* years (95% HPD)	*env* years (95% HPD)
SIVsm root to HIV-2 A	122.8 (57.2–199.9)	218.6 (86.1–369.6)	80.8 (27.7–151.1)
SIVsm root to HIV-2 B	126.2 (59.2–203.7)	227.5 (101.3–379.4)	76.1 (25.1–148.0)
SIVcpz root to HIV-1 M	293.8 (176.2–424.6)	529.4 (207.6–979.8)	402.8 (231.0–601.4)
SIVcpz root to HIV-1 N	347.2 (224.6–489.0)	666.0 (290.1–1225.6)	471.6 (291.6–693.2)
SIVcpz root to HIV-1 O	323.4 (202.0–458.2)	561.5 (229.0–1042.2)	413.5 (247.1–621.3)

## Discussion

The findings presented in this study indicate that the tMRCA of SIV in sooty mangabeys and chimpanzees is 1809 (1729–1875) and 1492 (1266–1685), respectively, assuming the relaxed molecular clock is unbiased. In addition, our results suggest that the time between the MRCA of SIVsm and SIVcpz and the MRCA of the human-to-human transmissible HIV lineages may be only hundreds of years. We present the tMRCA for all five of these HIV lineages, though we note that previous age estimates for HIV-1 groups M and O were based on larger datasets [5],[6]. We estimate the tMRCA for HIV-2 group A to be 1932 (1906–1955) and HIV-2 group B to be 1935 (1907–1961); these estimates were generated by incorporating a more biologically plausible model of rate variation among lineages, compared with the strict molecular clock used to obtain the previous HIV-2 tMRCA estimates [7].

In addition, we present the first date, to our knowledge, for the tMRCA of HIV-1 group N at 1963 (1948–1977). This date suggests that HIV-1 group N is the youngest transmissible HIV lineage and the only lineage to have originated in the second half of the twentieth century (though the possibility of a deeper history cannot be excluded given the sparse sampling). Taken together with the previous tMRCA estimates for HIV-1 groups M and O (circa 1900s and 1920s, respectively) and our updated HIV-2 group A and B dates (circa 1930s), it appears that SIV has given rise to transmissible HIV lineages throughout the twentieth century. The dispersed timing of these transmissions to humans implies that no single external factor is needed to explain the cross-species transmission of HIV. This observation is consistent with both of the two prevailing views of the origin of the HIV epidemics. The first is the bushmeat hypothesis [22], whereby SIV is transmitted to humans during the slaughter or butchering of infected primates. The second is that the growth of sub-Saharan African cities allowed for these nascent lineages to gain a foothold [5],[7]. According to the second hypothesis, SIV may have been jumping into humans since it first infected chimpanzees and sooty mangabeys. A change in human ecology then may have altered the evolutionary dynamics, whereby a virus that historically may have only infected a few individuals and then died out now has the potential to become an epidemic lineage. It does not seem farfetched to venture that SIV will continue to be transmitted to humans well into the twenty-first century.

**Figure 2:**
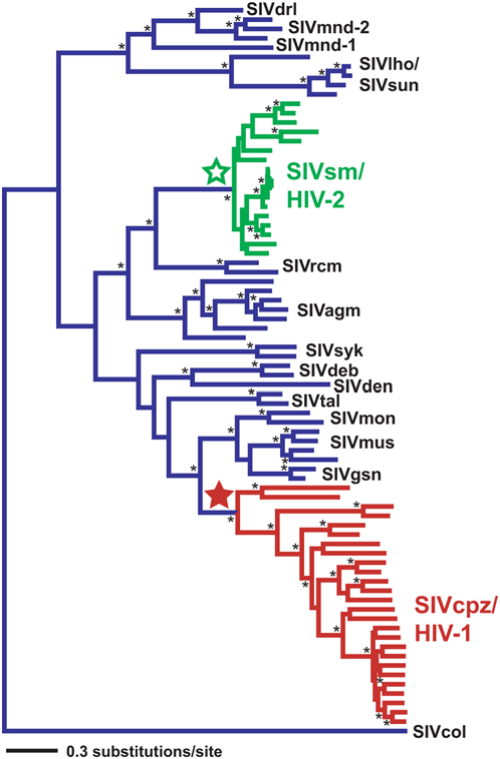
Maximum likelihood phylogeny of SIV/HIV *env* locus. SIVsm/HIV-2 lineages are green and their MRCA is designated with an open green star. SIVcpz/HIV-1 lineages are red and their MRCA is designated with a closed red star. All other SIV lineages are blue. Tree is midpoint rooted. Nodes with bootstrap support >80% are indicated with an asterisk.

There are several arguments suggesting that SIV has been present in sooty mangabeys and chimpanzees longer than our results indicate. First, coalescent processes or selective sweeps might have removed the deeper lineages from the phylogeny. While we cannot discount the latter, our finding that the SIVsm population has not evolved under a constant size suggests that deep SIVsm lineages may still be present. It remains unclear whether coalescent processes may have removed deep SIVcpz branches. Nevertheless, the full SIV/HIV tree suggests that there is a relatively short period of time between the MRCAs of SIVsm and SIVcpz and the branches that lead to SIVs that infect other primates (Figure 2). A second argument is that our sampling was not thorough enough, and deep SIV branches were not included. While possible, other studies that included additional non-dated SIVsm and SIVcpz sequences did not uncover additional deeper branches [8],[18],[23]. Thirdly, it has been suggested previously that SIV may lack the clock-like signal necessary to draw inference about tMRCAs. As a part of this study, we demonstrated that one major SIV lineage evolves in a clock-like fashion and at a rate indistinguishable from HIV. SIVsm sequences sampled over 30 years contain enough information to calibrate the molecular clock and date the tMRCA of an SIV clade. While we were able to use the SIVsm rate to date the tMRCA of SIVsm/HIV-2, this dating was not possible for SIVcpz. This difference is likely because we had far fewer SIVcpz sequences that were sampled over a relatively small window of time. Lastly, it is possible that our relaxed-clock models are biased and therefore unable to accurately date SIV coalescent events. We cannot dismiss this possibility, but the accuracy of these methods has been previously confirmed by other studies predicting the year of sampling of older HIV isolates from 1959 and 1960 [4],[5]. Furthermore, our analyses recovered HIV tMRCA estimates that are in line with those previously inferred for the age of the HIV clades. Conversely, if one were to accept the HIV dates, one would need to provide a compelling reason not to accept the tMRCA estimates for SIVsm and SIVcpz as well. If the SIV tMRCAs are not correct, then we would need to determine what would be biasing their estimates, because it might also be affecting the HIV tMRCAs and those of other RNA viruses.

The young ages of SIVsm and SIVcpz suggest that the entire SIV phylogeny may be relatively young (Figure 2). Even if SIV was present in sooty mangabeys and chimpanzees prior to the coalescence of their current diversity, we have identified divergence events deep in the SIV phylogeny that are on the order of hundreds of years old. The case of SIVsm is particularly compelling in this context since SIVsm sequences alone returned such a young date. It is difficult to reconcile these ages with an SIV phylogeny that is millions of years old. It seems more reasonable that the SIV phylogeny is on the order of thousands or tens of thousands of years old. While it had previously been suggested that a young-looking phylogeny could actually be the result of codivergence over millions of years, this argument was partly predicated on the assumption that SIV did not have a reliable clock-like signal [17]. In light of our findings, this argument is no longer tenable. What is still needed, however, is a reliable estimate of the age of the entire SIV phylogeny.

SIV is not the only virus once thought to be ancient whose phylogeny may be better explained by the preferential host switching model. Hantaviruses infect a wide array of rodent and insectivore species. Their phylogeny was thought to be the result of an ancient infection followed by codivergence, but recent evidence suggests that the virus and host phylogenies are too dissimilar to suggest codivergence [24]. Furthermore, the molecular clock in hantaviruses suggests a tMRCA orders of magnitude younger than that of their hosts [25]. In addition, the similarity of the Arenavirus phylogeny to that of its host may also be the result of preferential host switching [26]. Furthermore, it has been proposed that feline immunodeficiency virus, a lentivirus whose lack of associated disease in natural feline hosts was thought to be the result of an ancient infection, codiverged and coevolved with its feline hosts [27],[28]; however, in light of the possible young age of SIV, it may be worth taking a more detailed look at the relationship between the feline immunodeficiency virus and feline phylogenies.

Given the ages of the SIV clades presented here, it seems unlikely that SIV evolved apathogenicity over millions of years of coevolution and codivergence with its primate host species. It is still possible that SIV evolved avirulence in its natural hosts. If SIV were highly pathogenic when it first infected sooty mangabeys and chimpanzees, then it might have decreased in virulence over a remarkably short period of time, possibly on the order of hundreds of years. There remains the distinct possibility, however, that SIV was rarely pathogenic in its natural hosts and the low level of disease associated with SIV infection is actually the ancestral phenotype. The theory of ancient coevolution towards apathogenicity appears less plausible, given the recent discovery that SIVcpz is pathogenic in wild populations of the eastern chimpanzee subspecies (Rudicell RS, Jones JH, Pusey AE, Terio KA, Estes JD, Raphael J, Lonsdorf EV, Wilson ML, Keele BF, and Hahn BH. (2009) SIVcpz is pathogenic in its natural host. Oral Abstract. 16^th^ Conference on Retroviruses and Opportunistic Infections). Future work distinguishing between these two alternative theories on SIV apathogencity is needed.

A young age for SIV contrasts with other ancient retroviruses. The simian foamy virus appears to be at least 30 million years old, based on congruence between the viral and primate host phylogenies [29]. Furthermore, lentiviruses, the viral group to which SIV belongs, are also millions of years old, based on the presence of defective endongenous lentiviruses in rabbits and lemurs [30]–[32]. None of these findings, however, preclude the possibility that SIV is a much younger lentiviral clade.

Finally, it is possible that SIV itself is much older than the tMRCA of the extant lineages. Dating the tMRCA of influenza A viruses infecting avian hosts suggested that deep viral lineages were constantly lost, which resulted in younger than expected tMRCA estimates for subtypes [33]. A similar process of lineage birth and death may have occurred among SIV, in which SIVs infecting particular primate species would occasionally go extinct and later be replaced by a new species-specific SIV. This process would involve the loss of deep SIV lineages with replacement by younger ones. This extinction and reinfection would be analogous to the loss of deep branches due to the coalescent. If such a phenomenon operated across the entire SIV tree, it could mask the ancient age of the virus. Combined with a preferential host switching mechanism, a macro-evolutionary process such as this could account for a young tMRCA for an ancient virus whose phylogeny is similar to that of its host.

## Methods

### Sequences and alignments

SIVsm/HIV-2 (*gag*, *pol*, and *env* sequences) and SIVcpz/HIV-1 (non-recombinant full-length genome sequences) with sampling dates were obtained from the Los Alamos National Laboratory HIV sequence database (http://hiv.lanl.gov/content/index) (Table 5). The majority of the SIVsm sequences (>85%) were sampled from infected sooty mangabeys in US primate centers between 1975 and 2005 [18],[34]. Dated sequences from macaques infected with SIV from sooty mangabeys were also included. We excluded HIV-1 group M subtype G, as this lineage is likely of recombinant origin [35]. To prevent sampling bias from HIV-1 group M lineages, only two sequences, selected randomly, of each subtype from each year were included in the alignment. Sections of the SIVcpz/HIV-1 genomes that correspond to the *gag*, *pol*, and *env* regions used for the SIVsm/HIV-2 analyses were designated (Table 5).

**Table 5:** SIV and HIV alignments used in BMCMC analyses.

Taxa	Locus	Number of sequences	Length of sequences (nucleotides)	Range of sampling
SIVsm/HIV-2	*gag*	189	477	1975–2005
	*pol*	155	612	1975–2005
	*env*	181	438	1975–2005
SIVsm only	*gag*	166	477	1975–2004
	*pol*	134	612	1975–2005
	*env*	155	438	1975–2005
SIVcpz/HIV-1	*gag*	178	666	1983–2005
	*pol*	179	801	1983–2005
	*env*	178	582	1983–2005

To improve the accuracy of phylogenetic inference, we excluded (*i*) recombinant regions, determined using BootScanning in the RDP package [36],[37], (*ii*) multiple sequences from single individuals, (*iii*) sequences containing frame-shift mutations, and (*iv*) ambiguously aligned regions. Sequences containing frame-shifts were removed to accommodate codon-partitioning models in our phylogenetic analyses. Alignments were performed using Clustal X [38] and manually cleaned in Se-al (http://tree.bio.ed.ac.uk/software/seal/). SIVsm/HIV-2 and SIVcpz/HIV-1 alignments are provided as supporting information (Datasets S1, S2, S3, S4, S5, S6).

### Relaxed molecular clock analyses

To infer the tMRCA for the major SIVsm/HIV-2 and SIVcpz/HIV-1 lineages, we employed a BMCMC approach implemented in BEAST v1.4.7 [39],[40]. Initially, each of the three loci for both SIVsm/HIV-2 and SIVcpz/HIV-1 datasets was analyzed independently. Uninformative priors (i.e., tree priors) were placed on all internal nodes whose tMRCAs were estimated. We tested the appropriateness of GTR+Γ_4_ and SRD06 models; the latter allows for different Κ and Γ values for the third codon position [41]. Three different coalescent tree priors were investigated: constant population size, exponential growth, and Bayesian skyline plot. We compared the six different model combinations for each locus using Bayes factor in Tracer v1.4 (http://beast.bio.ed.ac.uk/Tracer). The Bayes factor provided strong support for SRD06 over GTR+Γ_4_ (Bayes factor>20), but there was not support for one coalescent scenario over any of the others. For SIVcpz, an exponential coalescent model produced substantially younger ages; this observation is not surprising given that a single exponential growth rate for SIVcpz and the HIV-1 group M pandemic lineage would likely underestimate the age of SIVcpz. The date estimates from the Bayesian skyline plot runs were used for both SIVsm and SIVcpz analyses because this model places the fewest constraints on the data [42]. XML input files for the SIVsm/HIV-2 *gag* and SIVcpz/HIV-1 *env* Bayesian skyline plot BMCMC runs are provided as supporting information (Datasets S7 and S8). Additional XML input files are available from the authors upon request.

Two BMCMC runs of 50 million generations were performed for each analysis to ensure convergence of parameter estimates. Tracer was used to check for convergence and mixing (estimated sample size>200). Trees were annotated using the maximum clade credibility tree. All analyses were performed using an uncorrelated lognormal relaxed molecular clock [39]. Each analysis was also run without data to better appreciate how the prior may be affecting the tMRCA estimates. The complete SIV/HIV phylogeny was constructed using a heuristic search in a maximum likelihood framework using a GTR+Γ_4_ model in PAUP* v4.1 [43]. Topological support was assessed using non-parametric bootstrapping (100 replicates using a heuristic search in a maximum likelihood framework). We used *env* instead of the entire SIV genome because many SIV lineages are of recombinant origin [44]. The *env* alignment used to construct this phylogeny was obtained from the curated Los Alamos National Laboratory sequence database.

To determine which locus's tMRCA estimates may be affected by a lack of demographic signal, we performed partition analyses. First, we pruned the SIVsm/HIV-2 dataset to contain only those sequences that were found in all three genes from the same individual and sampling year. In BEAST, we analyzed these reduced datasets assuming constant population size and exponential growth. We then concatenated all *gag*, *pol*, and *env* alignments, and each locus was partitioned to allow it to have its own tree topology, substitution model, relaxed clock model, and tMRCA estimates; they shared only the coalescent demographic parameter(s). This analysis was performed assuming constant population size or exponential growth. Partitioning was not possible for Bayesian skyline plot, as this model's demographic estimates are topology-dependant. The same protocol was used with the SIVcpz/HIV-1 dataset.

All date estimates provided are mean values with 95% HPD. Comparisons of tMRCA estimates among BMCMC runs (e.g., among loci and SIV/HIV versus SIV-only) were performed by asking how many times the estimate from one run was greater than the estimate from another run. This value was taken as the probability (P) that the two runs were different.

## Supporting Information

Dataset S1 SIVsm/HIV-2 gag nexus alignment.
(0.10 MB TXT)

Dataset S2 SIVsm/HIV-2 pol nexus alignment.
(0.10 MB TXT)

Dataset S3 SIVsm/HIV-2 env nexus alignment.
(0.08 MB TXT)

Dataset S4 SIVcpz/HIV-1 gag nexus alignment.
(0.13 MB TXT)

Dataset S5 SIVcpz/HIV-1 pol nexus alignment.
(0.15 MB TXT)

Dataset S6 SIVcpz/HIV-1 env nexus alignment.
(0.11 MB TXT)

Dataset S7 SIVsm/HIV-2 gag XML.
(0.16 MB TXT)

Dataset S8 SIVcpz/HIV-1 env XML.
(0.17 MB TXT)

Figure S1 Maximum clade credibility tree for HIV-1 group N using all available env sequences. Sequences from individuals not included in the initial analysis are indicated with a super-script a. The phylogeny was inferred using MrBayes v3.1. Posterior probability values (>0.9) are shown of nodes. Tree is rooted with the SIVcpz sequence that is sister to HIV-1 group N.
(0.50 MB PDF)
