# Supplementary figures and images for: Dating the Age of the SIV Lineages That Gave Rise to HIV-1 and HIV-2
Source: PLoS Comput Biol. 2009 May 1;5(5):e1000377. doi: 10.1371/journal.pcbi.1000377 (PMC2669881; doi:10.1371/journal.pcbi.1000377)

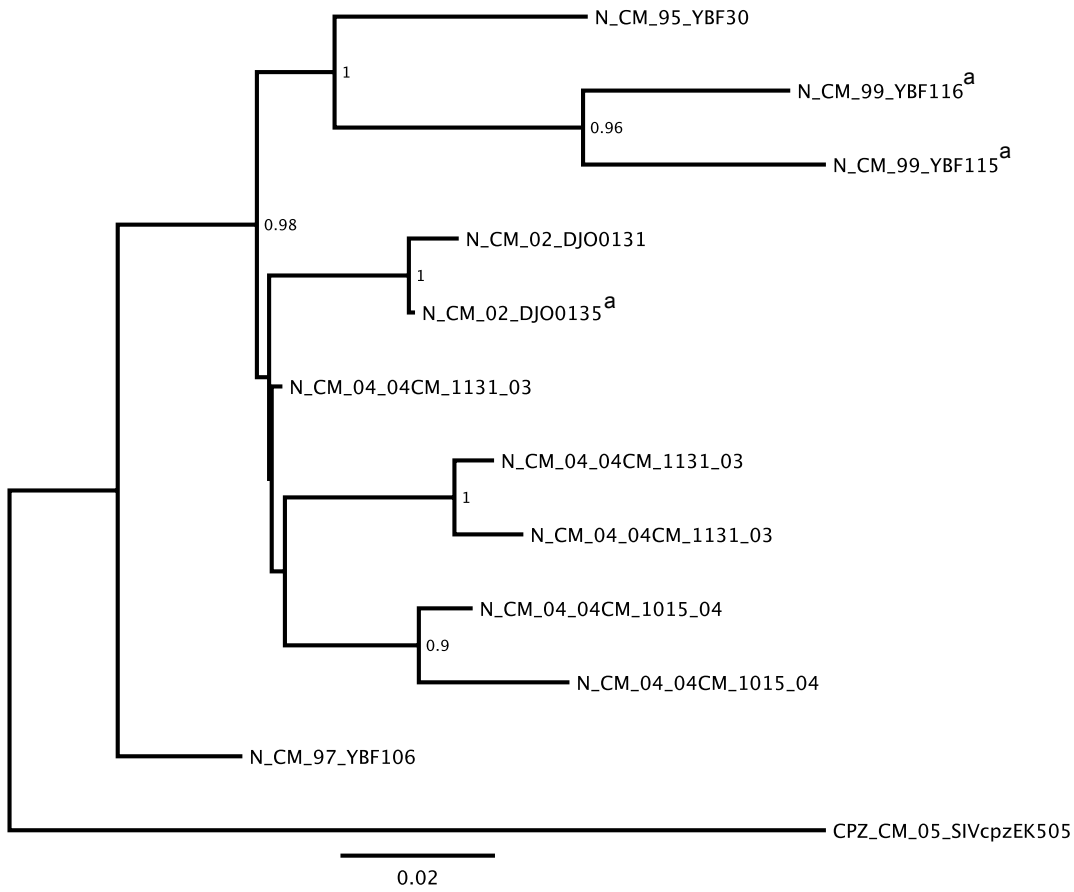

Supplement: Figure S1 — Maximum clade credibility tree for HIV-1 group N using all available env sequences. Sequences from individuals not included in the initial analysis are indicated with a super-script a. The phylogeny was inferred using MrBayes v3.1. Posterior probability values (>0.9) are shown of nodes. Tree is rooted with the SIVcpz sequence that is sister to HIV-1 group N. (0.50 MB PDF) [file pcbi.1000377.s009.pdf]
